# Early findings on home delivery of buprenorphine and retention in treatment for opioid use disorder

**DOI:** 10.1186/s13722-025-00545-2

**Published:** 2025-02-12

**Authors:** Marlene C. Lira, Lauren E. Hendy, Alisha Liakas, Laura Turanchik, Clare Pritchard, Cynthia Jimes, M. Justin Coffey

**Affiliations:** 1 Workit Health, 3300 Washtenaw Ave., Ste. 280, Ann Arbor, MI 48104 United States of America; 2 Health Policy, Johns Hopkins Bloomberg School of Public Health, Baltimore, MD 21205 United States of America; 3 Geisinger Commonwealth School of Medicine, Scranton, PA 18510 United States of America

**Keywords:** Opioid use disorder, Telemedicine, Buprenorphine, Medication delivery, Home delivery, Pharmacy barriers, Treatment retention

## Abstract

Individuals with opioid use disorder face barriers accessing first-line pharmacotherapy. Home delivery interventions have been shown to improve medication adherence for other chronic diseases, but the relationship between buprenorphine home delivery and opioid use disorder treatment outcomes has not been assessed. We evaluated the association between medication home delivery and retention in treatment in a feasibility study of adults who initiated telemedicine treatment for opioid use disorder and received one or more prescriptions. We described the characteristics of patients and estimated the odds of attending a telemedicine visit three and six months after enrollment as a function of home delivery use using logistic regression. The sample consisted of 337 adults with the following characteristics: mean age 40.8 years (SD 10.1), 51.0% male, and 70.9% commercially insured. In the first 30 days of treatment 6.8% (*n* = 23) of patients used home delivery. At three months, the percentages of individuals retained among those with and without home delivery were 82.6% and 58.9%, respectively (odds ratio [OR]: 3.31, 95% confidence interval [CI]: 1.10–9.96). At six months, the percentages of individuals retained among those with and without home delivery were 78.6% and 45.5%, respectively (OR: 4.39, 95% CI: 1.19–16.25, *n* = 203). Although uptake of medication delivery through the pharmacy partner was low within this sample of patients receiving treatment for opioid use disorder, its use was associated with increased retention in care at three and six months. Given the small sample size, low uptake, and limited statistical power, additional research is warranted.

## Background

Recent policy changes have reduced prescribing barriers for buprenorphine, including expanded telehealth prescribing flexibilities for controlled substances and the removal of the X-waiver, an opt-in certification that allowed healthcare providers to prescribe buprenorphine to a limited number of patients [1, 2]. Nevertheless, many individuals receiving treatment for opioid use disorder (OUD) continue to experience obstacles in obtaining prescribed medications [3]. Pharmacies face challenges from ordering limits on buprenorphine due to distribution oversight programs mandated in opioid settlement agreements [4]. Separately, pharmacists are hesitant to undergo audits from the Drug Enforcement Administration as a result of high volumes of buprenorphine dispensing [4]. Together, these barriers have led to constrained buprenorphine inventory in pharmacies. Stigma also contributes to medication access challenges with one in five pharmacies reportedly unwilling to fill buprenorphine prescriptions at all [5–7]. Individuals receiving telemedicine OUD treatment may encounter additional barriers: the geographic distance between the patient and their telehealth provider can trigger ‘red flag’ warnings for potential diversion risk in pharmacies’ dispensing computer systems. These red flags can lead to fill delays or denials [8].

Improving access to buprenorphine prescriptions could yield substantial public health benefit for individuals living with OUD. Past research has demonstrated the benefit of mail-order pharmacies on medication adherence in the treatment of hypertension, diabetes, high cholesterol, heart disease, stroke, and depression [9–11]. Home delivery has also improved access to naloxone nasal spray, an opioid antagonist used to reverse opioid overdose, through harm reduction initiatives that distribute the medication via mail [12]. However, in contrast to naloxone nasal spray, buprenorphine is a pharmacotherapy for the treatment of OUD that is generally taken long-term. To our knowledge, home delivery of buprenorphine has not been studied. We aimed to evaluate the feasibility of a buprenorphine home delivery pilot program to impact retention in telemedicine treatment for OUD.

## Methods

**Fig. 1:**
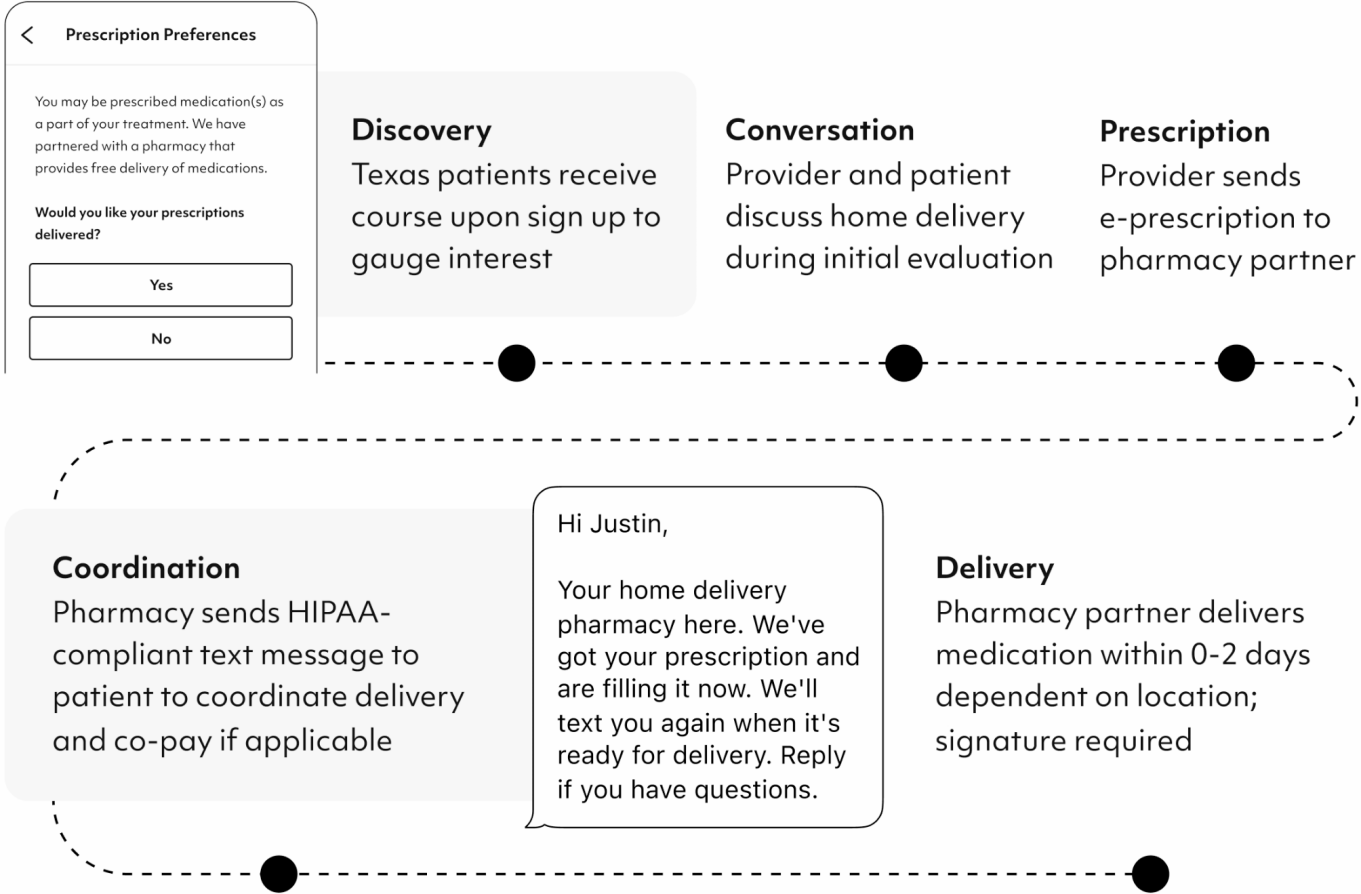
Operational workflow of buprenorphine home delivery within telehealth treatment for opioid use disorder

To address medication access for patients receiving treatment for OUD amid these systemic challenges, we partnered with a retail pharmacy in Texas offering mail delivery for buprenorphine prescriptions with same-day to 2-day shipping time (Fig. 1). Using data from the first eight months of the partnership, we conducted a retrospective cohort analysis to assess the preliminary impact of medication home delivery on retention in telemedicine OUD treatment. This study was approved through an expedited review by an external IRB (Solutions IRB; FWA00021831). Individuals were included in the analysis if they attended an initial visit for opioid use disorder at the Texas telemedicine clinic between May 1, 2023, and December 31, 2023, and received at least one prescription.

### Telemedicine model of care and home delivery intervention

The telemedicine model of care is available to individuals seeking treatment for OUD who have access to an internet-accessible device and reside in the state of the operating telemedicine clinic, and has been described previously [13]. In brief, patients connect through a phone-based app or website for video-based medical and behavioral health visits, counselor- and peer-led support groups, case management, virtual urine drug screens, and automated assessments as part of measurement-based care [13]. This model resonates with patients who face challenges to in-person care such as childcare or transportation barriers, or those who prefer the privacy of telemedicine compared to in-person office visits. The telemedicine practice focuses on substance use disorders and common comorbid conditions including depression, anxiety, hepatitis C, and insomnia. The pharmacy partnership was developed as part of a Small Business Innovation Research contract through the National Institute on Drug Abuse. The pharmacy partnership focused on the delivery of buprenorphine for OUD, although patients could choose to transfer additional prescriptions to the pharmacy as well. During the contract, investigators submitted a 513(g) request for information to the Food and Drug Administration, and the pharmacy partnership was determined to be exempt. The pharmacy sent text messages throughout the fulfillment process to coordinate co-pays and to provide additional information such as when the medication was en route for delivery and tracking information. Signatures were required upon delivery for controlled substances including buprenorphine.

Patients in treatment at the Texas telemedicine clinic were able to self-select into receiving buprenorphine through home delivery. Providers did not decide if a certain patient was appropriate for home delivery. Because individuals starting buprenorphine need to cease use of other opioids and experience withdrawal before starting the medication, the timing of the initial prescription is critical. To support patients who wanted to use home delivery but were not able to wait up to two days for a prescription to arrive, an initial prescription was commonly sent by their provider to a local pharmacy, and the home delivery pharmacy partner was activated for subsequent prescriptions.

### Statistical analysis

The exposure was having a prescription sent to the home delivery pharmacy partner within 30 days of the initial medical evaluation. The outcomes were three- and six-month retention in treatment, defined as attending an appointment between 68-113 days and 158–203 days (i.e., 45-day windows) after the initial medical evaluation, respectively. Six-month retention was assessed among a subgroup of individuals who initiated treatment at least 203 days prior to data analysis.

Patient age, gender, and rural residency were assessed during intake through self-report. Billing type was measured as a categorical variable representing Medicare, Medicaid, commercial coverage, or self-pay financing based on billing records. We described the sample using mean and standard deviation for age and frequencies and proportions for all categorical variables overall and by exposure status. Bivariate differences in utilization of home delivery were assessed by t-tests, chi-squared tests, or Fisher’s exact tests, as appropriate. The likelihood of retention in treatment at three and six months was modeled using logistic regression. Odds ratios were evaluated with a threshold for significance at 0.05. Analyses were conducted in Stata (Version 18, College Station, Texas).

## Results

The sample included 337 individuals with the following characteristics: 70.9% commercial insurance; mean age 40.8 years (SD 10.1); 51.0% male (Table 1). Twenty-three individuals (6.8%) used the home delivery pharmacy partner within the first 30 days of treatment. Individuals using home delivery were older and more likely to have Medicare coverage.

**Table 1:** Characteristics of patients initiating telemedicine treatment for opioid use disorder by home delivery use

Characteristic	Overall (*n* = 337)	Used Home Delivery Within First 30 Days (*n* = 23)	Did Not Use Home Delivery Within First 30 Days (*n* = 314)	*p*-value
**Mean age (SD)**	40.8 (10.1)	45.5 (8.4)	40.5 (10.2)	**0.021**
**Billing type**				
Commercial insurance	239 (70.9%)	17 (73.9%)	222 (70.7%)	**0.020**
Medicaid insurance	2 (0.6%)	1 (4.3%)	1 (0.3%)	
Medicare insurance	48 (14.2%)	5 (21.7%)	43 (13.7%)	
Self-pay financing	48 (14.2%)	0 (0.0%)	48 (15.3%)	
**Gender**				
Woman	161 (47.8%)	15 (65.2%)	146 (46.5%)	0.24
Man	172 (51.0%)	8 (34.8%)	164 (52.2%)	
Other	4 (1.2%)	0 (0.0%)	4 (1.3%)	
**Residence**				
Rural	13 (3.9%)	1 (4.3%)	12 (3.8%)	0.90
Urban	324 (96.1%)	22 (95.7%)	302 (96.2%)	

At three months, the percentages of individuals who were retained in care among those using and not using home delivery were 82.6% and 58.9%, respectively (odds ratio [OR]: 3.31, 95% confidence interval [CI]: 1.10–9.96). At six months, the percentages of individuals who were retained in care among those using and not using home delivery were 78.6% and 45.5%, respectively (OR: 4.39, 95% CI: 1.19–16.25, *n* = 203).

## Discussion

Within this sample of patients receiving treatment for OUD, utilization of a pharmacy partner offering home delivery of buprenorphine in the first 30 days of treatment was low. Approximately 7% of patients opted to use buprenorphine home delivery while a study of U.S. adults taking prescription medications found approximately 16% used mail-order delivery, most commonly for cardiovascular or diabetes medications [14]. However, due to fears regarding opioid craving and withdrawal, individuals initiating buprenorphine may be more concerned about the reliability of medication shipping compared to individuals using mail order for other chronic conditions.

Despite low uptake, utilization of the home delivery pharmacy was associated with over three times the odds of retention in care at three months and over four times the odds of retention in care at six months. These early findings suggest pharmacy home delivery can help reduce barriers to accessing buprenorphine and encourage long-term recovery efforts through continued engagement in OUD care. Over time, increased retention in OUD treatment should reduce the risk of opioid overdose and the impact of opioid-related mortality in communities. However, we note this analysis was limited by low statistical power and the potential for selection bias. The number of patients who opted into buprenorphine home delivery represented only a small fraction of patients eligible to use medication shipping and as a result, the confidence intervals around the odds ratios for treatment retention were wide. The small sample size also impacted our ability to conduct adjusted analyses to account for potential confounding. Patients in more stable recovery at the start of OUD treatment may have been more likely to use home delivery and if so, our findings may reflect their greater underlying propensity to be retained in treatment rather than the impact of buprenorphine home delivery. Additional research is needed to understand the impact of medication delivery on OUD treatment outcomes controlling for potential confounders. Randomized controlled trials of buprenorphine shipping and retrospective research with larger sample sizes are important to examine the association between buprenorphine home delivery and OUD treatment retention while accounting for characteristics such as age, gender, race/ethnicity, rurality, socioeconomic status, other medication use, and OUD recovery status. Exploratory qualitative research could also advance this emerging field by exploring patient and provider-level barriers and facilitators to the use of buprenorphine home delivery. Nevertheless, this feasibility study provides preliminary support that home delivery of buprenorphine may be a promising strategy to increase retention in treatment for opioid use disorder.

## Data Availability

No datasets were generated or analysed during the current study.

## Abbreviations

OUD: Opioid use disorder
